# The influence of extended fasting on thyroid hormone: local and differentiated regulatory mechanisms

**DOI:** 10.3389/fendo.2024.1443051

**Published:** 2024-08-26

**Authors:** Xiukun Sui, Siyu Jiang, Hongyu Zhang, Feng Wu, Hailong Wang, Chao Yang, Yaxiu Guo, Linjie Wang, Yinghui Li, Zhongquan Dai

**Affiliations:** ^1^ State Key Laboratory of Space Medicine, China Astronaut Research and Training Center, Beijing, China; ^2^ School of Life Science and Technology, Harbin Institute of Technology, Harbin, China

**Keywords:** long-term fasting, hypometabolism, thyroid hormone, extreme environment, deiodinase, tanycyte

## Abstract

The hypometabolism induced by fasting has great potential in maintaining health and improving survival in extreme environments, among which thyroid hormone (TH) plays an important role in the adaptation and the formation of new energy metabolism homeostasis during long-term fasting. In the present review, we emphasize the potential of long-term fasting to improve physical health and emergency rescue in extreme environments, introduce the concept and pattern of fasting and its impact on the body’s energy metabolism consumption. Prolonged fasting has more application potential in emergency rescue in special environments. The changes of THs caused by fasting, including serum biochemical characteristics, responsiveness of the peripheral and central hypothalamus-pituitary-thyroid (HPT) axis, and differential changes of TH metabolism, are emphasized in particular. It was proposed that the variability between brain and liver tissues in THs uptake, deiodination activation and inactivation is the key regulatory mechanism for the cause of peripheral THs decline and central homeostasis. While hypothalamic tanycytes play a pivotal role in the fine regulation of the HPT negative feedback regulation during long-term fasting. The study progress of tanycytes on thyrotropin-releasing hormone (TRH) release and deiodination is described in detail. In conclusion, the combination of the decrease of TH metabolism in peripheral tissues and stability in the central HPT axis maintains the basal physiological requirement and new energy metabolism homeostasis to adapt to long-term food scarcity. The molecular mechanisms of this localized and differential regulation will be a key research direction for developing measures for hypometabolic applications in extreme environment.

## 1 Introduction

Fasting, with a history spanning nearly thousands of years, has become an ideal lifestyle choice for purportedly maintaining physical and mental well-being, improving metabolic syndrome (1) and combating cancer (2), protecting from chemotherapy toxicity (3) and promoting longevity. Medical fasting for 2 days or more has been applied for preventive and therapeutic purposes in specialized centers (4). Additionally, long-term fasting (LTF) has long been known to induce hypometabolism and hypothermia in humans and animals (5, 6), and has significant potential in the physiological maintenance during emergency rescue, particularly in extreme environments such as mine disasters, cave explorations, and manned deep space explorations. Food deprivation will be last for a long time in such conditions. Reducing energy expenditure is the best approach to enhance survival and rescue efforts. However, the practical application of fasting interventions has become increasingly challenging due to the distinct and individual consequences of short-term complete fasting for up to two days in currently prevalent forms (7, 8) and the limited evidence-based clinical data of LTF (9). More information is necessary for its broader implementations as a therapeutic prescription for diseases or valuable survival strategies for extreme environments of prolonged fasting (10).

Fasting confers healthy benefits to a greater extent than just being attributed to a reduction of caloric intake and may involve a metabolic switch, enhanced autophagy, and antioxidant capacity, as well as cellular stress resistance (11, 12). Metabolic switching from liver-derived glucose to adipose-derived ketones as a fuel source has been observed and contributes to improvements in energy metabolic regulation. Recent studies show that individuals can well tolerate a 10-day complete fasting (CF), experience an energy metabolic substrate shift, and achieve new metabolic homeostasis between days 3 to 6 of CF, with a declining resting metabolic rate (13). Additionally, fasting significantly impacts the regulation of thyroid hormone (TH) levels, which play a crucial role in the regulation of energy metabolism. However, the influence of fasting on energy metabolism and TH metabolism varies depending on the duration and frequency of fasting (14). The physiological mechanisms involved in these effects are complex and required further exploration. The principles of fasting, modifications in TH metabolism, and possible regulatory mechanisms are outlined in the present review. Here we discuss the mechanisms of potential metabolic regulatory and biological effects, and ultimately provide effective practical methods for food conservation, as well as physiological and behavioral adaptations to endure long-term food limitations.

## 2 Fasting definition and patterns

In humans, fasting typically refers to the dietary pattern of intentionally abstaining from food and caloric beverages for periods ranging from 12 hours to 3 weeks (15), Generally, the body enters a fasting state 8-12 hours after the last meal. Many religious groups incorporate fasting into their rituals for spiritual development and health promotion, including Daoist Bigu, Buddhist fasting, and Muslim Ramadan fasting (RF) (16). Fasting therapy is an ancient folk health method that has been established as a defined therapeutic approach in modern medicine, particularly in specialized hospitals or clinical departments of integrative medicine. Following expert consensus in Germany (17) and China (18), fasting therapy has become an evidence-based preventive treatment for modern diseases. Essentially, fasting is a physiologically adaptive process that restricts energy intake. Under different fasting modes, the body constantly makes physiological metabolic adjustments, reduces energy requirements, and systematically utilizes its own energy reserves and repairs aging components through processes such as fat breakdown, and autophagy. This leads to an improvement in physiological state and a delay in senescence, helps to prevent and treat conditions like obesity, diabetes, and cardiovascular diseases (19). Different fasting patterns produce different physiological impacts, and the health-improving effects vary accordingly.

In general, fasting can be classified into four main modalities based on fasting duration and interval. 1) Caloric Restriction (CR): This involves typically decreasing daily caloric intake by 15% to 40%. 2) Intermittent Fasting (IF): Refers to a cycle of eating patterns that can range from 12 hours to several days, with recurring periods of little to no caloric intake. Time restricted feeding (TRF) and alternate day fasting (ADF) are forms of IF. TRF limits daily food intake to a 4-8 hours window, while ADF alternates normal food intake with days of restricted food intake. The religious fasting practices of RF fall into this category. 3) Long-term Fasting or prolonged fasting (LTF): This involves completely abstaining from food for more than 2 consecutive days, typically ranging from 3 to 21 days (20, 21). Certain forms of Daoist Bigu belong to this category. 4) Periodic Fasting (PF): Lasts 2 to 7 days periodically on a weekly, monthly, or yearly scale (22). The “5:2” diet, a popular fasting regimen, involves 5 days of ad libitum feeding and two fasting days per week, during which food intake is markedly decreased to approximately 500-600 kcal. These PF/IF protocols reduce energy intake for a period of time, repeatedly mobilizing and storing energy substrates. This results in more frequent but less pronounced alterations compared to continuous fasting (15). During LTF, physiological mechanisms ensure the survival of glucose-dependent tissues and employ alternative energy source to fuel other tissues. LTF is capable of deeply remodeling energy metabolism and causes stronger effects than CR and IF (13, 23). These characteristics highlight the therapeutic potential of LTF. Moreover, extended fasting can effectively reduce resting energy expenditure (EE) and has application potential in emergency rescue under special environment with food shortage, although the inducing efficiency to hypometabolism and the health maintenance mechanism need to be further explored.

## 3 Influences of fasting on energy metabolic expenditure

LTF fasting reduces BMR and EE, but the effects of short-term fasting and IF have not been concluded. Fasting is a popular controlled nutritional intervention for its potential benefits in weight loss and over health improvement. Understanding its effects on EE can help tailor fasting regimens and guide to practical applications. The effects of fasting on EE vary depending on the type of fasting utilized, its duration, frequency and refeeding method (24, 25). Under normal conditions, there is an equilibrium between energy intake and expenditure. EE occurs primarily through the resting metabolic rate (RMR, accounting for approximately 60-70% of total EE), followed by physical activity (contributing about 20-30%) and the thermic effect of food (responsible for roughly 10%of total EE) (26). First of all, the effects of short-term fasting or IF on EE are still being determined. It has been observed that resting EE briefly increased during the first few days of fasting, likely due to increased sympathetic nervous system activity and catecholamine release. Other contributions to increased EE may include the energy costs of fatty acid recycling and gluconeogenesis (31, 32). The resting EE was not different after 12 hours and 60 hours of fasting, but the proportional contribution of carbohydrate, fat and protein oxidation was significantly shifted (33). Total EE showed a decrease of -1.9 MJ/day, but resting EE remained unchanged in obese individuals after 6 days of fasting for weight loss (34). Heilbronn et al. observed no significant changes in RMR and RQ from baseline to day 21 of ADF, but there was an increase in fat oxidation (35). Although RF is associated with decreased activity and sleeping time, no significant alteration was observed in RMR or total EE (36). The daily total EE and resting EE decreased about 12.4% and 6.5% respectively, after a month of RF, but there was no statistical difference in the EE of physical activity (37). Some reports indicated that the change in RMR varies at different times of RF duration. RMR was higher in the first week of RF and showed a significant downward trend in subsequent weeks, potentially due to metabolic adaptation mediated both centrally and locally (36, 38). Therefore, while IF regimens and short fasting may initially boost EE, extended fasting and continuous CR prompt a proportional metabolic slowdown. However, LTF and severe CR have been demonstrated to lower RMR and result in metabolic adaptation (13, 27). As early as 1915, Benedict reported a 20-30% decrease in energy metabolism induced by prolonged starvation (28). LTF significantly decreases 24-hour EE and the respiratory exchange ratio, as detected by a respiration chamber (29, 30). The respiratory quotient (RQ) approaches 0.7 with the extension of fasting time in a 10-day CF experiment, indicating fat as the predominant fuel source (13). An important phenomenon is that fasting leads to a significant decrease in serum thyroid hormone T3, which may contribute to the decline in RMR (39). From an evolutionary perspective, animals instinctively consume more energy to find food in the initial stage of food deprivation (13). By the way, the increased EE in the early stage of food deficiency suggest that we should strengthen psychological guidance to reduce this consumption. This metabolic adaptation likely contributes to conserve energy and enhance survival during prolonged food scarcity such as under some extreme environments.

## 4 Impacts of fasting on thyroid hormone metabolism

### 4.1 Effects of fasting on TH levels

**Figure 1:**
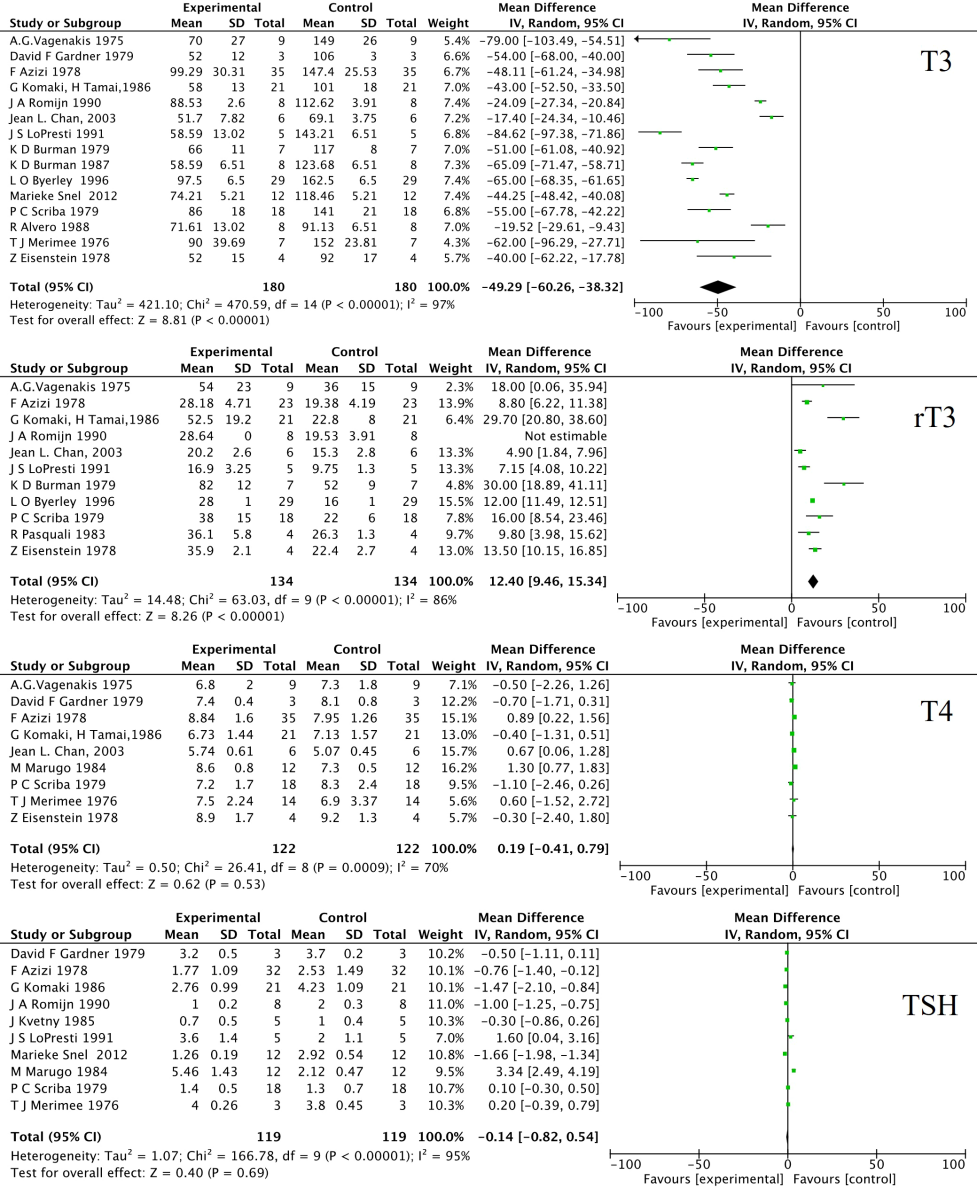
Forest plot of the effects of long-term fasting on T3, rT3, T4 and TSH. 16 human research papers were selected by manually review from PubMed and Web Of Science using the following term: “long-term fasting” or “prolonged fasting” and “thyroid hormone”. The data were analyzed by RevMan 5.3 for MacOS (Cochrane Collaboration, Oxford, UK) as the standard protocol and parameters. The weighted mean differences (WMD) were applied for the comparison of continuous variables. The heterogeneity in studies was assessed through the chi-squared (χ^2^) test and inconsistency index (I^2^). χ^2^ p value < 0.05 or I^2^ > 50% were considered as significant heterogeneity. A random-effect model was used to estimate the combined WMD. Otherwise, the fixed-effect model was applied. SD, standard deviation; IV, inverse variance; CI, confidence interval; df, degree of freedom.

As a crucial endocrine regulatory system for basal metabolic rate and EE, hypothalamus- pituitary-thyroid (HPT) axis is profoundly affected by various fasting protocols. To save energy and conserve protein during food shortages, one of the major adaptations is the down-regulation of TH-dependent metabolism. Firstly, the decrease in TH levels during fasting is usually temporary and reversible. Once the individual resumes normal diet habits, the TH levels return to normal. In some cases, LTF or severe CR can lead to a more significant decrease in TH and may even result in low T3 syndrome. Secondary, it is well known that fasting induces alterations of circulating THs, characterized by a marked decrease in serum triiodothyronine (T3), free T3 (FT3), an increase of reverse T3 (rT3) (40, 41), and inconsistent changes in thyroxine (T4), free T4 (FT4) and thyroid stimulating hormone (TSH), which may show a slight decrease or remain unchanged during fasting as depicted in **Figure 1** by a meta-analysis during LTF. A 24-hour short-time fasting decreases FT3 by 6%, but increases rT3 by 16% in healthy humans (42). Animal experiments with 12- to 48-hour fasting also confirmed the decreases of serum T3 and T4 as summarized in a recent review (43). Fasting did not alter plasma thyrotropin-releasing hormone (TRH) levels after a 6-day fasting (44). The concentration of TRH in both the median eminence (ME) and the hypothalamic portal blood decreases during fasting (45). As a typical negative feedback regulation, the HPT axis involves the synthesis and release of TRH in the hypothalamus, which stimulates the synthesis and secretion of TSH in the anterior pituitary. Then TSH promotes the thyroid to release T4, which is later converted to active T3 by deiodinase type I (Dio1) and type II (Dio2). In turn, excessive T3 and T4 inhibit the synthesis and secretion of both TRH and TSH. The decreased serum TH concentration was not accompanied by a rise of TSH or TRH during food deprivation, reflecting a deviation from the classic negative feedback regulation of the HPT axis.

Local TH metabolism is probably a determinant of net effects and presents a flexible and dynamic regulation, which plays an optimal role in the organism’s adaptation to a range of environmental challenges (46).The expression changes of TH metabolism-related genes differ in the liver, thyroid, pituitary and hypothalamus of mice after 48 hours of fasting (47). Differential regulations of TRH metabolism by fasting depend on the age, and species of the animals (48, 49). Thus, the combination of the decrease of TH metabolism in peripheral tissues and stability in the central HPT axis maintains the basal physiological requirement and new energy metabolism homeostasis to adapt to long-term food scarcity. The tissue variability in regulatory pattern is manifested in the differential responsiveness of the central and peripheral HPT axis to fasting, especially the intracellular T3 availability (50).

### 4.2 Fasting affects peripheral TH metabolism

During fasting, THs are shifted toward inactivated metabolism including the increase of rT3 conversation and decrease of T4 to T3 conversation. The decreased peripheral conversion of T4 to T3 is likely the primary mechanism responsible for circulating TH change, due to the lack of an appropriate TSH and TRH response in serum. As previously described, a prominent feature of the fasting state is a significant decrease in circulating T3 levels and an increase in rT3. Total caloric deprivation appears to shift the peripheral metabolism of T4 from the activation pathway to the deactivation pathway (51), possibly due to the deceased generation of T3 and the metabolic clearance rate of rT3 (52). The constancy of T4 levels throughout the fasting period by administration of L-thyroxine could not restore the decrease in serum T3, indicating that fasting decreases T3 production (41, 53). The enzyme activity for the conversion of T4 to T3 in fasted rat liver homogenate was reduced by 54%, primarily due to a reduction in enzyme concentration rather than co-factor availability (54). After a 48-hour fasting, only the transport of T3 into the perfused rat intracellular liver compartment decreased, but not the transport to the extracellular liver compartment (55). The clearance of rT3 is also affected by fasting. Both 7 and 13 days of fasting decreased rT3 clearance without changing rT3 production, compared to controls (56). However, Galton et al. found unchanged or even increased liver T3 and T4 concentrations within 16- or 36-hour fasting, with an increase in T3 clearance due to sulfation and UDP-glucuronidation (57). Some reports indicate that T3 glucuronidation was diminished, but T3 sulfation and subsequent deiodination were unaffected in the perfused rat liver after a 48-hour fasting (55). Similarly, Vries et al. also found that a 36-hour fasting did not alter intrahepatic T3 concentrations in rats (58), but a 48-hour fasting decreased both serum and liver T3 and T4 levels in mice (59). A gradual decrease of T3 and T4 concentrations in serum and liver during 12- to 48-hour fasting is confirmed in a recent review (43).

### 4.3 The potential alteration of central HPT response to fasting

Although fasting induces lower TH level, TSH and TRH secretion remains low, pointing to an altered hypothalamic setpoint of the HPT axis. Fasting, whether intermittent or prolonged, has complex effects on the response of the central HPT axis. The absence of an increase in serum TSH and TRH implies decreased negative feedback from the central part of the HPT axis. Although some reports suggest no change in serum TRH during fasting, LTF influences the secretion and function of TRH through mechanisms such as suppressing the activity of TRH neurons, reducing TRH gene expression, and inhibiting TRH secretion (45, 49). Previous animal studies have shown direct and indirect effects of decreased serum leptin contributing to the decrease of hypophysiotropic TRH neurons (40, 49). Leptin directly acts on hypophysiotropic TRH neurons that project to ME to regulate TSH production in the pituitary and regulates TRH neurons through pro-opiomelanocortin (POMC) and agouti-related peptide/neuropeptide Y (AgRP/NPY). However, recombinant methionyl human leptin only prevents the fasting-induced TSH decrease and pulsatility, but does not significantly affect the changes of the TH level (60).

Moreover, most studies report significant decreases in TSH response to TRH after fasting (60–62), but not all (63). Burman reported in 1980 that 10-day fasting impaired TSH secretion after TRH infusion (64). In the early phase (48 hours) of a 6-day fasting experiment, serum TSH still decreased in the control and TRH infused subjects (44). A 60-hour fasting decreases the mean 24-hour TSH concentration, associated with a decline in mean TSH amplitude, but not its frequency (65). Longer fasting results in a significantly lower TSH response than shorter fasting (66). Recently, Sinkó reported that fasting for 24 hours or 48 hours did not change TH action in the hypothalamic arcuate nucleus-ME region using TH action indicator mice, coupled with decreased expression of TSHβ and FT3 levels (67). Fasting promotes a 94% reduction in TSHβ mRNA expression in the pituitary (47), suggesting a decrease in the response of the pituitary to the low serum levels of T4. Fasting induces an increase of Dio2 expression in the hypothalamus, followed by an upregulation of local T3 content, which is critical to augment the suppression of the central HPT axis and avoid a negative feedback TH elevation (68). These data suggest that there are more complex and fine-tuned regulatory mechanisms of the HPT axis to maintain its stability in the brain during fasting.

## 5 The fine regulation of tanycytes on HPT axis during fasting

Tanycytes play a crucial role in maintaining the homeostasis of the central HPT axis during fasting by controlling the supply and metabolism of THs. Tanycytes are specialized ependymoglial cells in the third ventricle and are recognized as multifunctional players in energy metabolism through the organization of hormonal and nervous signals (69). Moreover, early studies indicated that Dio2 is absent in the PVN neurons (70). Additionally, it has been observed that a higher dose of T3 than the physiological level is necessary to reduce TRH expression in the PVN (71). This implies that the negative feedback loop involving T3 and TRH in the PVN may be less sensitive, allowing for a more nuanced regulation of the HPT axis.

### 5.1 Tanycytes regulate the bioavailability of THs to neurons

Acting as gatekeepers of the blood-hypothalamic barrier, tanycytes express the monocarboxylate transporter 8 (MCT8), OATP1c1 (organic anion-transporting polypeptide 1C1), and Dio2 enzyme. T4 is taken up from the cerebrospinal fluid into β2 tanycytes and deiodinated into the active form T3 by the highly expressed Dio2. Then, T3 diffuses to neighboring neurons, supplying almost 80% of the adult brain’s T3 (72–74). Interestingly, the modulator of cellular TH bioavailability µ-crystallin (CRYM), which has high affinity to T3 and T4, is relatively less expressed in β2 tanycytes than in other subtype tanycytes (75). This differential expression adjusts the time-course of T3 interaction with its receptor or the efflux of T3, and the transcription of target genes (72). TSH induces transcriptional regulation of TH-gatekeeper genes through the Tshr/Gαq/PKC pathway in tanycytes (76).

Axons from TRH-containing neurons of the paraventricular nucleus (PVN) project into the ME, where the neuron terminals make contact with the β2 tanycytic end feet (77). Increased circulating TH upregulates the expression of pyroglutamyl peptidase II (PPII), a highly specific TRH inactivating enzyme, in tanycytes and enhances the degradation of extracellular TRH in the ME through glial-axonal association (77). Stimulation of the TRH receptor I increases the intracellular calcium in tanycytes via the Gαq/11 pathway, which in turn increases the size of tanycyte end feet and the expression of PPII, moderating the release of TRH (78). Moreover, the expression of PPII in β2 tanycytes is also upregulated in response to elevated T3 levels, thereby forming a more refined negative feedback regulation. Thirdly, tanycytes are characterized by molecular signatures that sense and integrate nutrient/hormone signaling to modulate and maintain energy homeostasis (79). Indeed, β tanycytes form a “barrier” by expressing tight junction proteins and controlling the access of peripheral metabolites and hormones (80, 81). Many studies have described the metabolic sensor role of tanycytes for glucose, amino acids, leptin etc. Fasting can upregulate the facilitated glucose transporter and enhance blood-hypothalamus barrier plasticity through VEGF-dependent signaling (82, 83). Tanycytes also express receptors involved in the HPT axis and control TRH release (72, 78). Leptin has been identified as a key regulator of the central HPT axis by regulating pro-TRH expression in the PVN and could restore type III iodothyronine deiodinase (Dio3) expression in the liver (84, 85). Tanycytes regulate leptin’s entry into the hypothalamus and play an important role in the supply of THs via the expression of TH transporters and deiodinases (81).

### 5.2 Tanycytes mediate a central TSH-TSHR feedback loop

Tanycytes have the high number of Tshr transcripts (86). TSH released from pars tuberalis (PT) acts locally on tanycytic TSHRs, resulting in increased Dio2 expression and TH synthesis (87). Both hypothalamic tanycytes and pituitary PT-specific cells can respond to different photoperiods and regulate circulating TH levels (88). Photoperiod-dependent seasonal variations are integrated by tanycytes via the detection of the TSH released from the PT in the anterior pituitary gland (89). The PT-derived TSH is distinctly glycosylated compared with pars distalis-derived TSH. Furthermore, PT-TSH, detectable in the circulation, does not stimulate the thyroid gland (90).

Although our understanding of the functional roles of tanycytes in the regulation of the central HPT during fasting is lacking, current evidences suggest that these local regulatory mechanisms form a more refined negative feedback regulation of TH metabolism, potentially contributing to the homeostasis of the central HPT axis during fasting.

### 5.3 The effects of fasting on the TH metabolism in tanycytes

Local hyperthyroidism in the hypothalamus suppresses TRH release, which helps avoid excessive TRH response that could affect the body’s adaptability to fasting. Tracer kinetic studies have shown that serum T3 levels can accurately predict tissue T3 content and T3 signaling in most tissues, except the brain and pituitary gland (91). The uptake and conversion of THs are important for local T3 bioavailability and its action in the brain. Although MCT8 plays a pivotal role in the transfer of THs across the blood-brain barrier, fasting for 48 hours induces no significant alteration of MCT8 and monocarboxylate transporter 10 (MCT10) in the hypothalamus and pituitary (47, 92). MCT10 expression was found to increase upon fasting, whereas MCT8 expression remained unchanged (58). Conversely, fasting significantly induced MCT8 expression in the ependymal layer and PVN in mice (93). The impact of fasting on MCT8 expression levels and regional distribution requires further thorough investigation.

Fasting indeed impacts the expression and activity of Dio2 in tanycytes. Researchers observed a 2-fold increase in the expression of Dio2 mRNA in the mouse hypothalamus, but noted no change in the expression of Dio3 and Thrβ (47). In the initial phase of fasting, Dio2 mRNA expression and activity are robustly upregulated in tanycytes, leading to an increase in locally formed T3 and the suppression of TRH (68, 94). Coppola et al. observed that an appropriate induction of Dio2 activity during negative energy balance is dependent upon both leptin and glucocorticoid signaling (95).

Fasting influences the TRH release via tanycytes. Fasting temporarily increases the levels of PPII and Dio2 mRNA in tanycytes after 48 hours, followed by an increase of PPII activity in the ME and a partial reversion of the reduction in PVN pro-TRH mRNA levels and the number of TRH neurons. This delayed increase of ME PPII in fasted rats may facilitate the maintenance of the deep down-regulation of the HPT axis function (45).

## 6 Differential regulatory mechanisms

### 6.1 Different expression and activity of deiodinases in tissues

Despite the abundant clinical evidence, there is still a very limited understanding of how tissue-wide TH bioavailability and local TH action are regulated during development and fasting (96). Fasting decreases TH levels while keeping TSH concentrations unaltered, which is linked to local Dios expression and activity alterations in the brain hypothalamus and liver. The individual contributions of the three Dio isoenzymes to systemic and local TH provision are distinct during fasting and exhibit different regulatory mechanisms.

The main source of daily circulating T3 comes from the out ring deiodination of T4 by Dio1 and Dio2 in human. Thyroid gland contributes with about 20% of the daily T3 production, and the residual 80% is contributed outside the thyroid parenchyma, mainly from liver (97–99). The decrease in hepatic deiodination activity may be the primary factor for the decrease in circulating T3 during fasting. A 30-hour fasting decreases the activity of Dio1 in the liver and pituitary, with no changes in the kidney, and reduces the Dio2 activity in brown fat (57). However, fasting induces an increase in Dio2 expression in the hypothalamus (68). The decrease in Dio2 expression and activity was tissue-specific given that cerebral cortex Dio2 mRNA remains stable during fasting period, which leads to reduction in serum T3 levels, whereas serum T4 remains largely unaffected (98).

Dio3 is regarded as the major TH-inactivating enzyme catalyzing both T4 and T3 into rT3 and T2. Fasting results in markedly decreased serum T3 concentration coupled with a marked decrease in liver T3 concentration. Fasting also increases Dio3 mRNA expression mediated by the CAR and mTOR pathway in mice liver and WAT (59). A 36-hour fasting increases Dio3 activity, without changing Dio1 activity (14, 57). Dio3 plays a role in the fasting-induced alteration of TH homeostasis because the decrease of serum T3 is partially blunted in the Dio3-knockout mice after a 30-hour fasting (57). Leptin stimulates hypothalamus TRH expression and plays an important role in the regulation of the HPT axis. Fasting elicits significant reductions of serum leptin concentrations as shown by meta-analysis (100). Leptin administration restores the fasting-induced increase of hepatic Dio3 expression in mice, while serum TH levels and liver TRβ1 expression remain low (85). It is confirmed that fasting decreases the expression of Dio2 in the liver and increases it in the hypothalamus, and increases the expression of Dio3 in the liver. However, further exploration is needed to understand the expression and activity of Dios in the liver and hypothalamus, as well as the molecular mechanisms underlying their regulation.

### 6.2 Different expression of transporters in tissues

Concentrations of TH in local tissues are mediated by the uptake of circulating THs via their transmembrane transporters. The transport of T3 (55) and T4 (101) to liver cells is decreased, presumably due to the depletion of ATP in a perfused rat liver model. TH transport is mediated by widely expressed MCT8, MCT10 or brain-specific OATP1C1. Both MCT8 and MCT10 mediate T3 transport, but MCT8 also transports rT3 and T4, which are not efficiently transported by MCT10 (102). MCT10 mRNA expression is upregulated in the fasted liver, but MCT8 is not affected (14, 59). In mice, a 48-hour fasting induces a 4-fold increase of MCT8 mRNA expression in WAT, while MCT10 mRNA expression remains unchanged, indicating an enhanced uptake of T3 by adipocytes (59). Decreased MCT8 and increased MCT10 transcription in the fasted liver are also observed in rats. Moreover, the expression of both transporters is increased in the fasted gastrocnemius muscle (103). In the hypothalamus, the expression of MCT8 was reduced, but MCT10 did not change after 48-hour of fasting in mice, and its down-regulation upon fasting may be involved in this feedback mechanism (92). Till now, there is little data and research about the effects of fasting on OATP1C1, which is low expression in primates, so it is not discussed in present review.

## 7 Conclusion and perspective

Taken together, prolonged fasting has an application potential in emergency rescue under some special environments with food shortage, but the inducing efficiency to hypometabolism and the health maintenance mechanism still need to be understood, especially in the TH metabolism which plays an important role in energy expenditure regulation. Extended fasting significantly influences TH metabolism through localized and differentiated regulatory mechanisms in different tissues, involving the response of the HPT axis, reduced uptake and conversion of THs in the periphery, and nuanced regulation via tanycytes. The molecular mechanism of this local differentiation regulating deiodinated enzymes and transporters will be a top priority for future research.
